# Malnutrition and depression as predictors for 30-day unplanned readmission in older patient: a prospective cohort study to develop 7-point scoring system

**DOI:** 10.1186/s12877-021-02198-7

**Published:** 2021-04-17

**Authors:** Ika Fitriana, Siti Setiati, Edy W Rizal, Rahmi Istanti, Ikhwan Rinaldi, Taro Kojima, Masahiro Akishita, Muhammad Khifzhon Azwar

**Affiliations:** 1 Division of Geriatric Medicine, Department of Internal Medicine, Faculty of Medicine, Universitas Indonesia, Cipto Mangunkusumo Hospital, Jl. Diponegoro No.71, Salemba, Jakarta, Indonesia; 2 Clinical Epidemiology and Evidence Based Medicine Unit, Cipto Mangunkusumo Hospital, Faculty of Medicine, Universitas Indonesia, Jakarta, Indonesia; 3 Department of Geriatric Medicine, Graduate School of Medicine, The University of Tokyo, Tokyo, Japan

**Keywords:** Malnutrition, Depression, Comprehensive geriatric assessment, Predictive score, Readmission

## Abstract

**Background:**

Readmission is related to high cost, high burden, and high risk for mortality in geriatric patients. A scoring system can be developed to predict the readmission of older inpatients to perform earlier interventions and prevent readmission.

**Methods:**

We followed prospectively inpatients aged 60 years and older for 30 days, with initial comprehensive geriatric assessment (CGA) on admission in a tertiary referral centre. Patients were assessed with CGA tools consisting of FRAIL scale (fatigue, resistance, ambulation, illness, loss of weight), the 15-item Geriatric Depression Scale, Mini Nutritional Assessment short-form (MNA-SF), the Barthel index for activities of daily living (ADL), Charlson Comorbidity Index (CCI), caregiver burden based on 4-item Zarit Burden Index (ZBI), and cognitive problem with Abbreviated Mental Test (AMT). Demographic data, malignancy diagnosis, and number of drugs were also recorded. We excluded data of deceased patients and patients transferred to other hospitals. We conducted stepwise multivariate regression analysis to develop the scoring system.

**Results:**

Thirty-day unplanned readmission rate was 37.6 %. Among 266 patients, 64.7 % of them were malnourished, and 46.5 % of them were readmitted. About 24 % were at risk for depression or having depressed mood, and 53.1 % of them were readmitted. In multivariate analysis, nutritional status (OR 2.152, 95 %CI 1.151–4.024), depression status (OR 1.884, 95 %CI 1.071–3.314), malignancy (OR 1.863 95 %CI 1.005–3.451), and functional status (OR 1.584, 95 %CI 0.885–2.835) were included in derivation of 7 score system. The scoring system had maximum score of 7 and incorporated malnutrition (2 points), depression (2 points), malignancy (2 points), and dependent functional status (1 point). A score of 3 or higher suggested 82 % probability of readmission within 30 days following discharge. Area under the curve (AUC) was 0.694 (*p* = 0.001).

**Conclusions:**

Malnutrition, depression, malignancy and functional problem are predictors for 30-day readmission. A practical CGA-based 7 scoring system had moderate accuracy and strong calibration in predicting 30-day unplanned readmission for older patients.

## Background

Unplanned readmission is a well-known deleterious problem in older adults. The absence of a transitional care service system will add to the complexity especially considering case-mix national insurance regulation [1]. Under Medicare insurance coverage in the United States, the readmission rate was about 17.5 % [2]. In Indonesia, a country with a case-mix national insurance system, however, the readmission rate among older adults of a tertiary referral hospital was 20 % in 2008 [3]. Readmission is related to high cost, high burden, and high risk for mortality [4]. A scoring system can be developed to predict the readmission of older to perform earlier interventions and prevent readmission.

Older people have specific characteristics called geriatric syndromes which are often undetected by conventional medical approaches. Comprehensive geriatric assessment (CGA) has been shown to improve outcomes in this population [5]. One study demonstrated the role of Oncologic Acute Care for Elders (OACE) prognostic units to predict readmission by utilising an index of activities of daily living (ADL) index as one of the predictors for 30-day unplanned readmission [6]. Other scoring systems were developed for all ages, with area-under-the-curve (AUC) scores around 0.445 to 0.69 after validation [7]. A previous study used a 6-item Geriatric Brief Assessment (GBA) to predict 1-year readmission with C score of 0.58–0.61 [8]. Frailty [9], excessive polypharmacy[10], and caregiver burden[11] were previously suggested to be risk factors for readmission among older adults. Despite the existing evidence, few studies have explored the role of CGA in a scoring system to predict 30-day unplanned readmission in the older adult population.

 We aimed to develop an applicable predictive scoring system for medical wards based on CGA to predict all-cause 30-day unplanned readmission. We explored the role of malnutrition as one of the preventable geriatric syndromes and considered other geriatric conditions, including frailty, functional problems, depression risk, comorbidities, polypharmacy, and caregiver burden.

## Methods

### Study design and participants

The aim of this prospective cohort study was to develop a predictive scoring system for 30-day readmission based on CGA. We recruited all consecutive patients aged ≥60 years who were admitted to the acute care ward of Cipto Mangunkusumo Hospital, the national general hospital of Indonesia, from June to September 2019.

We collected the baseline data and 1-month follow up data after discharge from all patients who agreed to participate. The data collection was performed by three trained physicians, not including the researchers and authors of this study. Patients who died or were transferred to other hospitals were excluded from the study. The sample size was determined based on the equation for the sample size of prognostic studies [12]. All methods performed in this study were carried out in accordance with the relevant guidelines and regulations [13]. The study protocol was reviewed and approved by the Research Ethics Committee at the Faculty of Medicine, Universitas Indonesia.

### Baseline data collection and follow‐up

Unplanned readmission in this study was defined as readmission to the emergency room within 1 month after discharge. The baseline data used in this study relied on primary data collected with questionnaires. The data related to length of hospitalization and polypharmacy were collected from medical records. The data collected from the direct interviews were: (i) demographic data (sex, age, educational background, marital status, living status, caregiver, income); (ii) frailty status based on the Fatigue, Resistance, Ambulation, Illness, and Loss of weight (FRAIL) scale, the results of which were interpreted as normal (score 0), prefrail (score 1–2), and frail (score 3–5); (iii) functional status based on the Barthel ADL index questionnaire, the results of which were classified into totally dependent (score 0–4), severely dependent (score 5–8), moderately dependent (score 9–11), mildly dependent (score 12–19), and independent (score 20); (iv) nutritional status based on Mini Nutritional Assessment short-form (MNA-SF), the results of which were further classified into malnutrition (score 0–7), at risk of malnutrition (score 8–11), and normal (score 12–14); (v) cognitive status based on the Abbreviated Mental Test (AMT) questionnaire with three result classifications, namely severe cognitive impairment (score 0–3), mild cognitive impairment (score 4–7), and normal (score 8–10); (vi) depression status based on the 15-item Geriatric Depression Scale (GDS-15), the results of which were classified into normal (score < 5), strong probability of depression (score 5–9), and depression (score ≥10); (vii) comorbidity index based on the Charlson Comorbidities Index (CCI), the results of which range from mild (score 0–1), moderate (score 2–4), to severe (score ≥5); (viii) the amount of prescribed medication obtained from medical records; (ix) caregiver burden based on the 4-item Zarit Burden Index (ZBI), the results of which were interpreted as either no burden (score < 8) or burden present (score ≥8); (x) history of previous admission in the previous 6 months according to interviews with patients or family; (xi) severity of diseases based on case-mix severity level criteria according to ICD-10 and ICD-9 CM as planned by the national insurance obtained from our electronic medical records and documented as mild, moderate, or severe; (xii) the length of hospital stay was classified into 1–14 days or > 14 days; and (xiii) the presence of malignancy.

Following the discharge, we contacted the patients by phone and traced them with electronic health records on a weekly basis until 1 month (30 days) to collect their readmission status in any hospital.

### Statistical analysis

The incidence of 30-day unplanned readmission in this study was obtained by calculating the proportion of subjects who had experienced readmission within 1 month after discharge from the total number of subjects. For analytical purposes, the independent variables were categorized into dichotomous variables. Sex group was categorized into (1) male and (2) female. Age group was categorized into (1) ≤70 years old and (2) > 70 years old. History of admission in the previous 6 months was categorized into (1) no and (2) yes. Diagnosis of malignancy was categorized into (1) no and (2) yes. Frailty status was categorized into (1) fit to pre-frail and (2) frail. Functional status was categorized into (1) independent (mildly dependent or independent) and (2) dependent (totally, severely, or moderately dependent). Nutritional status was categorized into (1) normal and (2) at risk of malnutrition or malnourished. Cognitive status was categorized into (1) no cognitive problem and (2) cognitive problem (mild to severe cognitive impairment). Depression status was categorized into (1) normal and (2) strong probability of depression and depression. Comorbidity index was categorized into (1) mild to moderate and (2) severe. Number of prescribed drugs was categorized into (1) not having polypharmacy and (2) polypharmacy (consumption of ≥5 drugs on a daily basis) [14]. Caregiver burden was categorized into (1) no burden and (2) with burden. Severity of diseases was categorized into (1) mild to moderate and (2) severe. For statistical purposes, individuals with delirium and aphasia were considered as having higher risk for depression based on the evidence of previous studies [15–17].

The descriptive analysis was performed by calculating the proportion of all variables. A bivariate analysis using the chi-square test was applied to obtain the association between independent variables and 30-day unplanned readmission. A multivariate analysis using stepwise multiple logistic regression was performed for all variables with *p*-values < 0.25 in the bivariate analysis to obtain the prognostic factors for 30-day unplanned readmission.

We developed a predictive scoring system using the identified prognostic factors to predict 30-day unplanned readmission. Scores were obtained through stepwise calculation as follows: (1) dividing each prognostic factor’s coefficient B by its standard error (coefficient B/SE = x), (2) choosing the lowest x value as a reference (3) dividing each x value by the reference, and (4) picking the circled number nearest to the result from step (3). To evaluate the performance of the scoring system, we analysed the calibration and discrimination of the scoring system. The former used the Hosmer–Lemeshow test, whereas the latter used C-statistics and was described by AUCs and receiver-operating characteristics. The calibration and discriminating performance were validated by bootstrap resampling method for internal validity. Afterwards, the scoring performance was identified by using the Hosmer–Lemeshow test and C-statistics from the results of a repeated backward logistic regression model for each of the predictors of 1000 bootstrap resampling. The data were analysed using SPSS software (version 20; IBM SPSS, Armonk, NY, USA). The reported *p*-values in this study were two-sided and statistical significance was identified by a *p*-value < 0.05.

## Results

**Fig. 1:**
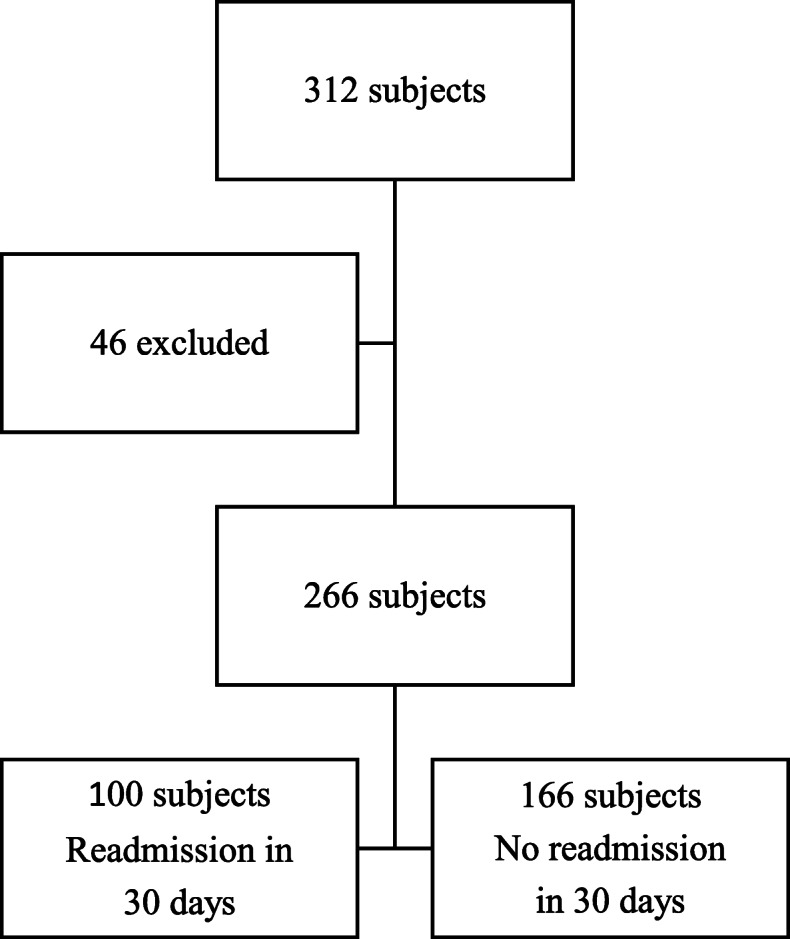
Flowchart of subjects included in study. Exclusion criteria: died at hospital or referred to other hospital (46 subjects died in the hospital), inclusion criteria: discharged from hospital (266 subjects)

We recruited 312 subjects who agreed to participate in this study. Forty-six subjects died during their hospitalization. All other subjects were discharged into the community (none of them went to a chronic care or social care service facilities) and could be followed up until the end of the study. The incidence of readmission rate in this study was 37.6 % (Fig. 1).

Table 1 presents the characteristics of the subjects. Among the older adults readmitted within the 30-day follow-up, most were women. About three out of five subjects were aged 60–69 years, whereas subjects aged ≥80 years only contributed to 7.9 % of the total number of readmitted older adults. There were 62.4 % married subjects and 55.3 % lived with their spouse, while only 4.9 % lived alone. Nearly all subjects had a caregiver who spent < 8 h per week taking care of the subject, with their spouse and children being the two most common caregivers. Among all subjects, 55.1 % still had income more than the average income for retirement age (3 million IDR per month). About three quarters of the subjects had a length of stay < 14 days.

**Table 1:** Baseline characteristics of subjects based on 30-day unplanned readmission status

Characteristics	30-day readmission	
	No, n (%)	Yes, n (%)
Sex		
Male	85 (65.9)	44 (34.1)
Female	81 (59.1)	56 (40.9)
Age		
60-69 years	104 (64.2)	58 (35.8)
70-79 years	49 (59)	34 (41)
≥80 years	13 (61.9)	8 (38.1)
Educational background		
No schooling	7 (70)	3 (30)
Elementary school	22 (53.7)	19 (46.3)
Junior high school	26 (60.5)	17 (39.5)
Senior high school	59 (58.4)	42 (41.6)
Higher	52 (73.2)	19 (26.8)
Marital status		
Unmarried	2 (40)	3 (60)
Married	111 (66.9)	55 (33.1)
Widowed	50 (55.6)	40 (44.4)
Divorced	3 (60)	2 (40)
Living status		
Alone	8 (61.5)	5 (38.5)
With spouse	97 (66)	50 (34)
With children	55 (58.5)	39 (41.5)
With others	6 (50)	6 (50)
Caregiver		
With caregiver	158 (62)	97 (38)
Without caregiver	8 (72.7)	3 (27.3)
Income (IDR)/month		
<1 million	17 (65.4)	9 (34.6)
1-3 million	52 (56.5)	40 (43.5)
More than 3 million	96 (65.3)	51 (34.7)
Length of stay (days)		
1-7	68 (66)	35 (34)
8-13	58 (61.7)	36 (38.3)
>14	40 (58)	29 (42)
Previous admission (in the past 6 months)		
No	103 (69.1)	46 (30.9)
Yes	63 (52.9)	56 (47.1)

Data are presented as proportion number and percentage of subjects for each variable. Total subjects *n*=266

*IDR* Indonesian Rupiah

### Derivation of risk score system for 30-day unplanned readmission

#### Predictors for 30-day unplanned readmission

The results of the bivariate analysis identified seven predictors for readmission (i.e. frailty status, functional status, nutritional status, cognitive status, depression status, malignancy diagnosis, and previous admission) (*p* < 0.05; see Table 2). Of the subjects, 44 % were frail and had a significantly higher risk of readmission than that for fit or pre-frail subjects. Subjects with poor functional status (needed assistance) also had higher risk for readmission. In this study, 35.2 % of the subjects were not malnourished nor at risk for malnutrition, while 35.6 % were at risk for malnutrition and 29.2 % were malnourished (MNA SF score 0–7). Among those subjects who had malnutrition or were at risk for malnutrition, 46.5 % were readmitted compared with only 21.3 % of normal subjects.

**Table 2:** Logistic regression analysis of independent variables according to 30-day readmission status (*n*=266)

Variable	Readmission status		RR (95% CI)	*p* value
	No, n (%)	Yes, n (%)		
Age				
≤ 70 years	113 (62.1)	69 (37.9)		
> 70 years	53 (63.1)	56 (40.9)	0.987 (0.834–1.167)	0.875
Sex				
Male	85 (65.9)	44 (34.1)		
Female	81 (59.1)	56 (40.9)	1.114 (0.925-1.343)	0.255
Length of stay (days)				
1-14	126 (67.3)	71 (36)		
>14	40 (58)	29 (42)	1.069 (0.919–1.244)	0.377
Frailty status				
Fit or prefail	105 (67.3)	51 (32.7)		
Frail	61 (55.5)	49 (44.5)	1.214 (0.994–1.483)	0.049*
Functional status (Barthel Index of ADL)				
Independent	76 (73.1)	28 (26.9)		
Dependent	90 (55.6)	72 (44.4)	1.315(1.098–1.576)	0.004*
Nutritional status (MNA-SF)				
Normal	74 (78.7)	20 (21.3)		
At risk for malnutrition or malnutrition	92 (53.5)	80 (46.5)	1.472 (1.236–1.752)	<0.001*
Cognitive status (AMT)				
Normal	129 (64)	67 (34.2)		
Cognitive problem	37 (52.9)	33 (47.1)	1.245 (0.976–1.588)	0.055*
Depression status				
Normal	124 (70.1)	53 (29.9)		
Strong probability or depression	42 (47.2)	47 (52.8)	1.485 (1.168–1.887)	<0.001*
Charlson Comorbidity Index				
<5 (not severe)	42 (59.2)	29 (40.8)		
>5 (severe)	124 (63.6)	71 (36.4)	0.930 (0.746–1.160)	0.509
Polypharmacy				
No	64 (61)	41 (39)		
Yes	102 (63.4)	59 (36.6)	0.962 (0.793–1.167)	0.693
Caregiver burden				
No burden	29 (56.9)	22 (43.1)		
With burden	137 (63.7)	78 (36.3)	1.189 (0.828–1.707)	0.363
Severity of disease				
Mild to moderate	93 (63.3)	54 (36.7)		
Severe	73 (61.3)	46 (38.7)	1.031(0.854–1.245)	0.748
Malignancy				
No	137 (66.8)	68 (33.2)		
Yes	29 (47.5)	32 (52.5)	1.406 (1.062–1.861)	0.006*
Previous admission (in the past 6 months)				
No	103 (69.1)	46 (30.9)		
Yes	63 (52.9)	56 (47.1)	1.284 (1.052–1.567)	0.011*

*RR* relative risk, *CI* Confidence interval, *ADL* activities of daily living, *MNA-SF* mini nutrition assessment-short form, *AMT* abbreviated mental test, *FRAIL* Fatigue, Resistance, Ambulation, Illness, Loss of weight

*Variables with *p*<0.25 were selected for the multivariate analysis

Depression status significantly contributed to the readmission rate. Subjects with cognitive problems also had a higher readmission status than those without cognitive problems, but this result was at the margin of statistical significance (47.1 % vs. 34.2 %, *p* = 0.055). Subjects with underlying malignancy also had significantly higher readmission rates than those without malignancy (52.5 % vs. 33.2 %, *p* = 0.006). Of the total number of subjects, 44 % (119 subjects) had previous admission in the past 6 months. Among those subjects, 47.1 % (56 subjects) had readmission. However, we found no significant difference between the readmission and readmission-free group for other variables, such as CCI, polypharmacy, and severity of disease (*p* > 0.05).

#### Stepwise multivariate regression analysis: developing 7-point scoring system

**Fig. 2:**
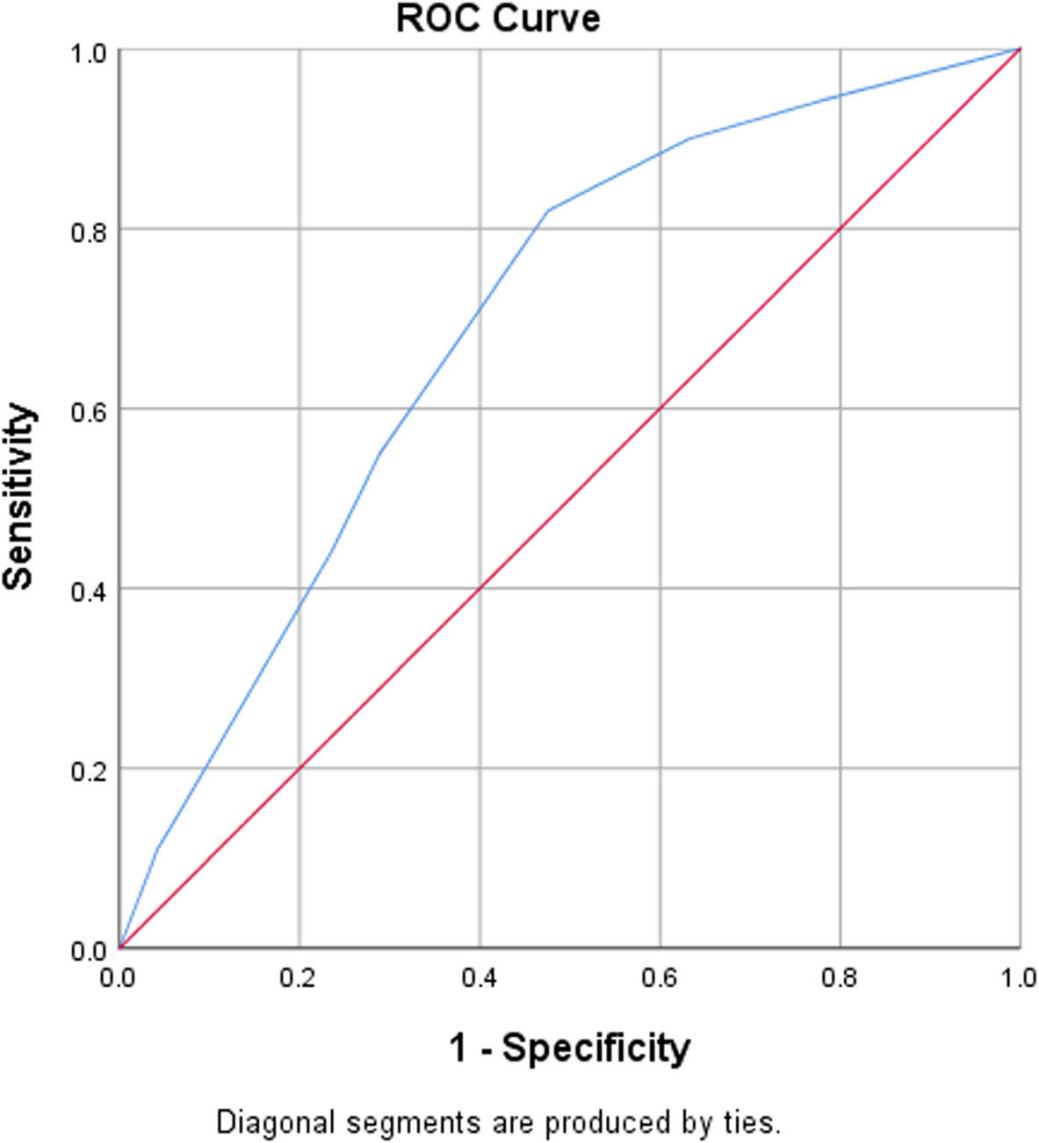
ROC (Receiver Operating Characteristics) for 7 score system as predictor for 30-day unplanned readmission in elderly population. Area under the curve (AUC) was 0.694, *p* < 0.05, Hosmer-lemeshow test 0.462

We identified seven variables with *p* < 0.05 for stepwise multivariate logistic regression (i.e. frailty status, functional status, nutritional status, depression status, cognitive status, malignancy diagnosis, and previous admission) (see Table 3). In the final model, four variables were identified in the development of a scoring system (i.e. depression status, nutritional status, malignancy diagnosis, and functional status) (see Table 4). Despite the insignificant difference in the last stepwise analysis for functional status, we decided to include this variable in the scoring system based on the evidence from previous studies [5, 18]. The AUC (Fig. 2) were higher when functional status was included (0.694) than when it was not included (0.681). We developed a 7-point scoring system (where functional status = 1, depression status = 2, cancer = 2, and malnutrition = 2) and put its results into bootstrapping logistic regression with Hosmer–Lemeshow score (*p* = 0.287, AUC = 0.694). The sensitivity and specificity table showed that a score ≥ 3 had 82 % sensitivity for predicting the risk for readmission.

**Table 3:** Odds ratio for independent variables for 30-day readmission status identified in multivariate logistic regression analysis (*n* = 266)

Variables	OR (95 % CI)	*p* value
Functional status (The Barthel ADL Index/ point)	1.647 (0.884–3.068)	0.116
Malignancy (Yes = 1, No = 0)	1.850 (0.975–3.571)	0.060
Nutritional status (MNA-SF/ point)	2.011 (1.072–4.113)	0.030
Depression status (GDS-15/ point)	1.878 (1.024–3.445)	0.042
Cognitive status (AMT/ point)	0.997 (0.508–1.954)	0.992
Frailty status (FRAIL scale/ point)	0.834 (0.455–1.528)	0.556
Previous admission in the previous 6 months (Yes = 1, No = 0)	1.370 (0.793–2.365)	0.259

*OR* odds ratio, *CI* confidence interval

**Table 4:** Derivation of 7-point scoring system to predict 30-day unplanned readmission from stepwise multivariate analysis (*n* = 266)

Variable	Coefficient B	SE	*p* value	OR (95 % CI)	Score
Depression status: Depression or strong probability of depression	0.633	0.288	0.028	1.884(1.071–3.314)	2
Malignancy diagnosis: Yes	0.622	0.315	0.048	1.863(1.005–3.451)	2
Nutritional status: At risk of malnutrition or malnourished	0.766	0.319	0.016	2.152(1.151–4.024)	2
Functional status: Dependent	0.460	0.297	0.121	1.584(0.885–2.835)	1

Data from stepwise multiple regression analysis, Adjusted OR 95 %CI. *SE* standard error, *OR* odds ratio

## Discussion

The readmission rate was 37.6 % in this prospective cohort study. Malnutrition, depression, malignancy, and dependent functional status were used as predictors for 30-day readmission, although the dependency-related finding was not significant in this study. The CGA-based 7-point predictive scoring system had moderate accuracy and strong calibration in predicting 30-day unplanned readmission for older patients.

The lengths of stay for the subjects in this study, irrespective of their 30-day readmission, were not similar to the recent Indonesian national data in 2020. The mean length of stay of inpatients aged ≥60 years in Indonesia was 5.94 days. Among all older inpatients nationwide, only 5.31 % required inpatient care for more than 14 days [19]. We believed that the subjects in our national referral hospital had more severe illnesses than the general older inpatients in Indonesia and thus required longer inpatient care. Most of our subjects also had severe comorbidities, which may be typical for older inpatients in a referral hospital [20] and can be related to higher readmission [21].

A study of older patients in France suggested a similar readmission rate in the setting of acute care for older adults (30.7 %) [8]. Similarly, the readmission rate among older people with malignancy was 35.2 %. [6]. A systematic review suggested that 30-day readmission rate of older people in single-center studies ranged between 11.7 and 30.0 %, whereas the range was 9.6–14.2 % in a multicenter study [22]. The higher readmission in our study might result from comorbidity and the severity of disease of the patients in addition to the lack of a comprehensive discharge planning and post-acute care system [23]. The insignificant result related to severe comorbidities may indicate that other factors contribute to the readmission.

We suggested that the results of CGA components (i.e. depression and nutritional and functional statuses), can be used as predictors for readmission. The finding of this study supported a suggestion from previous studies regarding depression [24, 25]. The results supported the finding of a study among older adults in the United States, in which high depressive symptoms were identified as significant risk of hospital readmission within 30 days following adjustment of other covariates. The odds ratio (OR) for the strong probability of depression or depression in our study was similar to that of a previous study (OR, 1.884; 95 % confidence intervals [CI]: 1.071–3.314 vs. OR, 1.66; 95 % CI: 1.01–2.74) [25]. Subjects with or at risk for depression had significantly higher risk to be readmitted, in part due to the possible lower compliance after discharge in subjects with depressive symptoms. Depression was also associated with delayed recovery in mobility or self-care. Despite the difficulties in collecting depression-related data during hospitalization, the study results suggest that subjects without depression were less likely to be readmitted.

In this study, we put the subjects at heightened risk for depression and those who were unable to complete the interview (i.e. individuals with delirium and aphasia) into one group for statistical analysis. We classified the aphasic individuals as being at risk for depression based on the finding of a previous study showing higher risk for depression in aphasic post-stroke patients compared to those without aphasia (47.5 % vs. 29.1 %, *p* < 0.01) [15]. The grouping method was also supported by the finding of a previous study suggesting that 70 % of aphasic patients met depression criteria during follow up assessment after cerebrovascular events [16]. Delirium may also be linked to depression with overlapping pathophysiology [17].

Interestingly, the nutritional problem was the strongest predictor among other factors to predict 30-day readmission, which was similar to the findings of other studies [26, 27]. Following the stepwise multivariate analysis, nutrition can also be included in our predictive scoring system. Malnutrition and readmission in a previous study were strongly related, but no studies include malnutrition in particular as a predictor among other geriatric syndromes [5, 28]. Malnutrition is a preventable geriatric syndrome, where interventions can be made despite the primary diagnosis and other health problems in hospitalized and post-acute care older adults. Malnutrition may be linked to the inflammation and loss of muscle mass, as described in the pathogenesis of phenotype frailty [29, 30].

Other factors, such as malignancy [31] and previous admission [32, 33] were previously suggested to be related to the readmission rate. A previous study in older adults suggested that malignant solid tumour was significantly associated with 10 times higher risk for hospital admission [34]. The admission and past oncological histories can be easily obtained from the medical record. Initially, both variables were significant predictors prior to adjustment for possible confounders in our study. However, multivariate analysis results suggested that the role of previous admission as predicting factor for readmission was not statistically significant.

The bivariate analysis results for polypharmacy, severity of comorbidity (assessed using CCI), and severity of disease were not significant, possibly due to various factors. Our finding related to polypharmacy supports the result of a previous study, in which polypharmacy (daily administration of ≥5 medications) did not significantly increase the risk of readmission of older adults in Sweden. The previous study found the significant increase in risk of readmission only in older adults with excessive polypharmacy, defined as taking ≥10 drugs on a daily basis (OR, 1.66; *p* = 0.007) [10]. Polypharmacy was more related to very early readmission (day 0–1 post-discharge) than risk factors for 30-day readmission [35]. The result of multivariable analyses from two studies also suggested that the CCI score was not significantly associated with higher risk of hospital readmission among older persons [34, 36].

Frailty status may have an impact on readmission [9, 37], whereas the FRAIL scale has a good predictive index to predict outcome (mortality and disability) in clinical settings [38]. However, the FRAIL scale result was not significant in the multivariate analysis of our study. The reason may be because frailty was correlated with other variables, such as malnutrition, depression, or functional status, which have stronger predictive scores. Likewise, the cognitive impairment result was also insignificant in the multivariate analysis. This finding may result from the link between cognitive function and depression status, which had a stronger prediction value. Several studies have suggested an association between cognitive status and the lower risk for readmission [39]. In contrast, other studies have also suggested the opposite results [40, 41], leading to conflicting evidence. Caregiver burden was also not a risk factor in this study, which differed from a previous study showing that caregiver burden was a risk factor for readmission [11]. Most caregivers in this study have a care burden, probably because patients admitted to the hospital had an acute condition following multiple comorbidities.

The finding related to functional status was not significant in this study, and may in part be due to its link to nutritional problems [42] and underlying depression [29, 43]. However, because there was a strong correlation with readmission and functional level was considered in the previous scoring system to predict readmission in older adults [6, 7, 44], functional status was included in the score. The inclusion of functional status score resulted in higher AUC.

The 7-point scoring system covers three geriatric syndromes, namely functional problem, depression, and malnutrition, with one disease entity (malignancy). This scoring system may predict 30-day unplanned readmission with moderate discrimination score (C-statistics, 0.694), good calibration (Hosmer–Lemeshow test, 0.462), as well as good internal validity for calibration (Hosmer–Lemeshow test, 0.287; *p* < 0.01), with score > 3 had 82 % probability that an older inpatient may be readmitted within 30 days. Many factors and the complexity of each geriatric health problem may contribute to the difficulties in developing a scoring system with good discrimination. This practical scoring system may be used in hospitals by health-care workers, such as general practitioners or geriatric medicine consultants, to potentially predict their patients’ readmission risk during hospitalization. This prediction would be followed by allocating specific interventions for high-risk patients that are both clinically effective and cost-effective.

To the best of our knowledge, this study is the first study to propose a practical 7-point scoring system to predict the risk of 30-day hospital readmission among older adults by using the multidimensional tools incorporated in CGA. However, we also acknowledge the limitations of this study. First, this is a single-centre study in a university-based hospital, which may be unsuitable for application in health-care centres taking care of older inpatients with fewer and milder comorbidities. Second, this study had relatively lower sensitivity as a scoring system with moderate C-statistics. Third, the initial examinations were performed in the acute phase, which might not describe the patient’s pre-discharge condition. These limitations may affect the results because the CGA results might change during inpatient care [3]. Future studies may need to follow the changes in CGA values regularly to understand the relationship between the CGA results and readmission rates.

## Conclusions

The components of CGA can be used to predict readmission and should be applied in all older patients, especially in those with multiple severe comorbidities. The role of nutritional intervention and prevention of depression in the hospital and post-acute care may be crucial to reduce the risk of readmission. This practical 7-point scoring system potentially predicts readmission while the patient is still hospitalized to allocate specific interventions and find ways for better management of high-risk patients.

## Data Availability

The datasets used and/or analysed during the current study are available from the corresponding author on reasonable request.

## Abbreviations

AUC: Area under the curve
GBA: Geriatric Brief Assessment
CGA: Comprehensive geriatric assessment
OR: Odds ratio
MNA-SF: Mini Nutritional Assessment short-form
ZBI: Zarit Burden Index
ADL: Activities of daily living
IADL: Instrumental Activities of Daily Living
GDS: Geriatric Depression Scale
CI: Confidence Interval
